# Major Vault Protein Inhibits Porcine Reproductive and Respiratory Syndrome Virus Infection in CRL2843^CD163^ Cell Lines and Primary Porcine Alveolar Macrophages

**DOI:** 10.3390/v13112267

**Published:** 2021-11-12

**Authors:** Xiaoping Wu, Junyang Fang, Qiuping Huang, Xu Chen, Zhongyi Guo, Lingyujia Tian, Enmin Zhou, Jianxin Chen, Yang Mu, Taofeng Du

**Affiliations:** 1 Department of Preventive Veterinary Medicine, College of Veterinary Medicine, Northwest A&F University, Yangling 712100, China; wz2012011713xp@163.com (X.W.); 18729396400@163.com (J.F.); Pinghezhiqiu87@nwafu.edu.cn (Q.H.); cx2474501747@163.com (X.C.); g18231705780@163.com (Z.G.); tlyj0118@nwafu.edu.cn (L.T.); zhouem@nwsuaf.edu.cn (E.Z.); 2 Guangdong Provincial Key Laboratory of Veterinary Pharmaceutics Development and Safety Evaluation, College of Veterinary Medicine, South China Agricultural University, Guangzhou 510642, China; jxchen@scau.edu.cn

**Keywords:** PRRSV, MVP, host factor, type Ⅰ interferon, antiviral

## Abstract

Porcine reproductive and respiratory syndrome (PRRS), a significant viral infectious disease that commonly occurs among farmed pigs, leads to considerable economic losses to the swine industry worldwide. Major vault protein (MVP) is a host factor that induces type Ⅰ interferon (IFN) production. In this study, we evaluated the effect of MVP on PRRSV infection in CRL2843^CD163^ cell lines and porcine alveolar macrophages (PAMs). Our results showed that MVP expression was downregulated by PRRSV infection. Adenoviral overexpression of MVP inhibited PRRSV replication, whereas the siRNA knockdown of MVP promoted PRRSV replication. In addition, MVP knockdown has an adverse effect on the inhibitive role of MVP overexpression on PRRSV replication. Moreover, MVP could induce the expression of type Ⅰ IFNs and IFN-stimulated gene 15 (ISG15) in PRRSV-infected PAMs. Based on these results, MVP may be a potential molecular target of drugs for the effective prevention and treatment of PRRSV infection.

## 1. Introduction

Porcine reproductive and respiratory syndrome virus (PRRSV) is a single-stranded, positive-sense, enveloped RNA virus which belongs to the Arteriviridae family. The length of the PRRSV genome is about 15 kb and encodes viral non-structural proteins as well as structural proteins [1]. Based on the divergence of gene sequences, PRRSV is divided into two genotypes, type 1 (PRRSV-1) and type 2 (PRRSV-2). In addition, the highly pathogenic PRRSV (HP-PRRSV) is a variant of type 2 PRRSV [2]. Pigs are the only natural host of PRRSV. The infection resulting from this pathogen causes a disease characterized by reproductive failure in sows as well as respiratory symptoms in newborn piglets and growing pigs [3,4], which has considerable economic consequences.

PRRSV infection is difficult to control. The evasion and suppression of host antiviral immune responses resulting in persistent PRRSV infection is thought to be one of the main reasons for this [5]. Firstly, PRRSV has evolved escape mechanisms in response to host innate immune responses. These mechanisms include inhibiting type Ⅰ interferon (IFN) responses [6], upregulating interleukin-10 (IL-10) [7,8], downregulating tumor necrosis factor α (TNF-α) [9,10], inducing apoptosis [11,12], and utilizing microRNAs (miRNAs) to evade host innate immunity [13,14]. Secondly, PRRSV has the ability to evade host adaptive immune responses by impairing PRRSV antigen presentation [15,16,17], activating immunosuppressive regulatory T cells (Tregs) [18], and utilizing antibody-dependent enhancement (ADE) [19,20].

Major vault protein (MVP), an evolutionarily highly conserved protein, is the major component of the largest cellular ribonucleoprotein particles, which are named vaults. Expression of MVP alone could form vault-like particles, which suggests that MVP is the main structural component of vaults [21]. In addition to MVP, vaults also include poly (ADP-ribose) polymerase, telomerase-associated protein-1 (TEP1) and small untranslated vault RNAs. MVP is mainly present in the cytoplasm of mammalian cells, and a small part was confirmed to be localized at or around the nuclear envelope and nuclear pore complex [22,23,24]. Previous studies have shown that MVP is responsible for various functions [25], such as multidrug resistance in anti-cancer drug treatment [26], nucleocytoplasmic transport [22,27], cellular signal transduction [28,29,30], cell differentiation [31], preimplantation embryo development [32,33], malignant transformation and tumor progression [34,35], autophagy [36], senescence [37] and innate immunity [38]. In antiviral immune responses, MVP interacts with myeloid differentiation primary response 88 (MyD88), leading to the production of type Ⅰ IFNs [39]. Recent studies have shown that the infection of several viruses upregulates MVP expression, including hepatitis C virus (HCV), vesicular stomatitis virus (VSV), influenza A virus (IAV), enterovirus (EV71), hepatitis B virus (HBV) and human immunodeficiency virus (HIV) [24]. Furthermore, overexpression of MVP inhibits the replication of HCV, VSV, IAV and EV71 by inducing type Ⅰ IFNs [40]. Hepatitis B virus (HBV) infection suppresses MVP–mediated type Ⅰ IFN production in vitro [39]. In HIV-infected monocyte-derived macrophages, HIV replication was promoted by the interaction between cystatin B and MVP [41].

In this study, the effect of MVP in PRRSV infection was evaluated in vitro. Our results showed that the downregulation of MVP expression was induced by PRRSV infection. Overexpression of MVP inhibited PRRSV replication; additionally, PRRSV replication was promoted in MVP-knockdown cells. MVP produced an anti-PRRSV effect by inducing type Ⅰ IFNs. Comprehensive results indicated that MVP expression regulation is a potential strategy against PRRSV infection.

## 2. Materials and Methods

### 2.1. Cell Cultures, Viruses and Chemicals

Porcine alveolar macrophages (PAMs) were isolated from healthy four-week-old PRRSV-negative pigs, as described previously [42]. CRL2843^CD163^, an immortalized cell line expressing the porcine ^CD163^ gene, is permissive for PRRSV infection [43]. PAMs or CRL2843^CD163^ cells were maintained in RPMI-1640 medium supplemented with 10% fetal bovine serum (FBS). MARC-145 cells were maintained in Dulbecco’s modified Eagle’s medium (DMEM) supplemented with 10% FBS. All cells were cultured at 37 °C and 5% CO_2_. All animal work was conducted strictly in accordance with the guidelines of the Institutional Animal Care and Use Committee and approved by the Animal Care and Use Committee of Northwest A&F University (Yangling, China).

Three highly pathogenic PRRSV isolates, GD-HD (GenBank: KP793736.1, Montgomery, MD, USA), VR-2385 (GenBank: JX044140.1), NADC30-like HNhx (GenBank: KX766379) and a PRRSV-1 isolate (GZ11-G1; GenBank: KF001144.1) were used in this study, which were propagated and titrated in MARC-145 cells.

Ruxolitinib, an inhibitor of Janus kinase (JAK) 1/2, inhibits JAK-signal transducer and activator of transcription (STAT) signaling pathway [44] (Topscience, Shanghai, China).

### 2.2. Overexpression of MVP and PRRSV Infection

The recombinant adenovirus plasmids carrying the MVP gene (Adv–MVP) were constructed by GENEWIZ. Briefly, the porcine MVP gene was cloned into shuttle plasmid CMV-MCS-SV40-EGFP. Subsequently, the recombinant shuttle plasmid and adenovirus backbone plasmid pBHG loxΔE1, 3 Cre (Microbix, Mississauga, ON, Canada) were co-transfected into HEK-293 cells to generate Adv–MVP. To produce a high-titer viral stock, the cell culture supernatants with Adv–MVP were concentrated by ultrafiltration and then purified using Adeno-X™ Virus Purification Kit (BD Biosciences Clontech, Palo Alto, CA, USA). The titers of purified viruses were detected by the end-point dilution assay. CRL2843^CD163^ cells or PAMs were seeded onto 12-well plates or 24-well plates and infected with the Adv–MVP at a multiplicity of infection (MOI) of five for 24 h or 12 h, followed by infection with PRRSV (at an MOI of 1 or 0.1). CRL2843^CD163^ cells or PAMs were harvested for Western blot analysis at 48 h post-infection (hpi) or 24 hpi, respectively. Cell supernatants were used for virus titration. Adenovirus plasmids without the MVP gene (Adv–Vector) were used as a negative control.

### 2.3. Knockdown of MVP and PRRSV Infection

CRL2843^CD163^ cells were transfected with specific siRNA targeting MVP (siRNA–MVP) at a final concentration of 100 nM using X-tremeGENE siRNA Transfection Reagent (Roche, Basle, Switzerland) following the manufacturer’s protocol. Lipofectamine^®^ RNAiMAX Transfection Reagent (Thermo Fisher Scientific, Waltham, MA, USA) was used to transfect siRNA–MVP in PAMs. The sequences of the siRNA–MVP are as follows: 5′–CCACUCCCAUCAACCUCUUTT–3′ (sense) and 5′–AAGAGGUUGAUGGGAGUGGTT–3′ (antisense). Twelve or thirty-six hours after transfection, CRL2843^CD163^ cells or PAMs were infected with PRRSV (at an MOI of 1 or 0.1), respectively. Then, cells were analyzed with qPCR and Western blot, and cell supernatants were harvested for virus titration. A non-targeting siRNA (NC–siRNA) was used as a negative control.

### 2.4. Quantitative Reverse Transcription-PCR (qRT-PCR)

Total RNA was extracted from PAMs using RNAiso reagent (TaKaRa, Dalian, China) and subjected to reverse transcription using PrimeScript RT Master Mix (Perfect Real Time; TaKaRa, Dalian, China), according to the manufacturer’s instructions. Quantitative PCR (qPCR) was conducted using a StepOnePlus^®^ Real-Time PCR System (Applied Biosystems, Foster City, CA, USA) with FastStart Universal SYBR Green Master (Roche, Basle, Switzerland). GAPDH was used as an internal reference. The primers used for qPCR are listed in Table 1.

### 2.5. Western Blot Analysis

The expression levels of MVP and the PRRSV N protein were analyzed by Western blot. Total cell lysates from CRL2843^CD163^ cells or PAMs were separated by 12% SDS-PAGE and transferred onto polyvinylidene difluoride (PVDF) membranes. The membranes were probed with monoclonal antibodies against MVP (Santa Cruz Biotechnology, Dallas, TX, USA), monoclonal antibodies against the PRRSV N protein (6D10, produced in our laboratory), or monoclonal antibodies against α-tubulin (TransGen Biotech, Beijing, China). HRP-conjugated goat anti-mouse IgG was used as the secondary antibody. Protein bands were visualized using Pierce™ ECL reagent (Thermo Fisher, Waltham, MA, USA).

### 2.6. Virus Titration

The titers of virus progeny from the supernatants of CRL2843^CD163^ cells or PAMs were detected by titration as described previously [45]. Briefly, MARC-145 cells were seeded into 96-well plates and incubated for 24 h. PRRSV-infected cell culture supernatants were 10-fold serially diluted (10^−1^–10^−7^) and added to each well (100 μL/well). Seven days after virus infection, the 50% tissue culture infection dose (TCID_50_) was calculated using the Reed–Muench method.

### 2.7. Statistical Analysis

Each experiment was repeated at least three times. The data were analyzed using GraphPad Prism version 5.0 (GraphPad Software, Inc., San Diego, CA, USA). Statistical differences were evaluated with Student’s *t*-tests for two groups or one-way analysis of variance (ANOVA) for more than two groups. *p* < 0.05 (two-tailed) was considered statistically significant.

## 3. Results

### 3.1. PRRSV Infection Inhibits MVP Expression in PAMs

**Figure 1:**
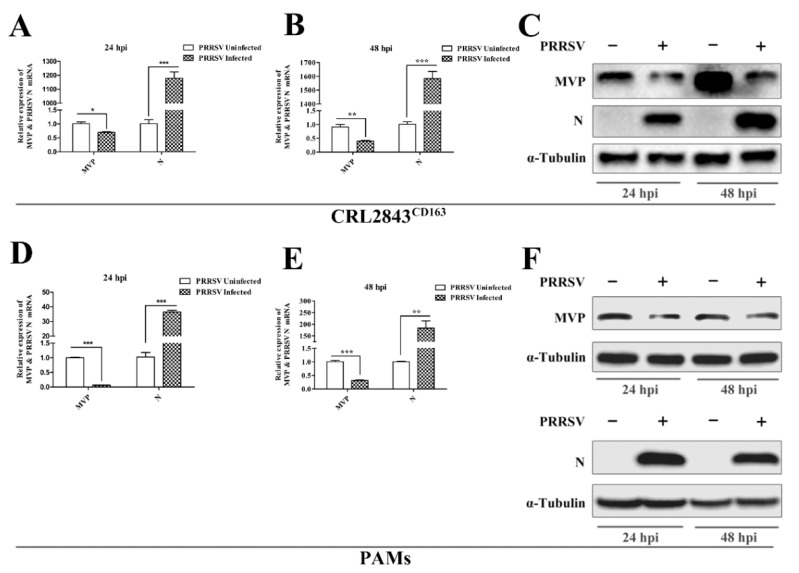
MVP expression was inhibited by PRRSV infection in CRL2843^CD163^ cells and PAMs. CRL2843^CD163^ cells or PAMs were infected with GD-HD PRRSV at an MOI of 10 or 1 for 24 h and 48 h. The mRNA and protein expression levels of MVP and PRRSV N were detected by qPCR (**A**,**B**,**D**,**E**) and Western blot (**C**,**F**), respectively. GAPDH and α-tubulin were used as internal loading controls in qPCR and Western blot analyses, respectively. Data from three independent experiments are expressed as the means ± SD. Analysis of differences between the means was performed using Student’s *t*-test and is marked by * (*p* < 0.05), ** (*p* < 0.01) and *** (*p* < 0.001).

To investigate whether MVP expression levels are affected by PRRSV infection, CRL2843^CD163^ cells or PAMs were infected with the GD-HD PRRSV strain, respectively. At 24 and 48 hpi, MVP mRNA and protein levels were detected by qPCR and Western blot, respectively. The results showed that the levels of MVP mRNA (Figure 1A,B,D,E) and protein (Figure 1C,F) expression were reduced by PRRSV infection at both time points when compared with the uninfected control.

### 3.2. Overexpression of MVP Inhibits PRRSV Replication In Vitro

**Figure 2:**
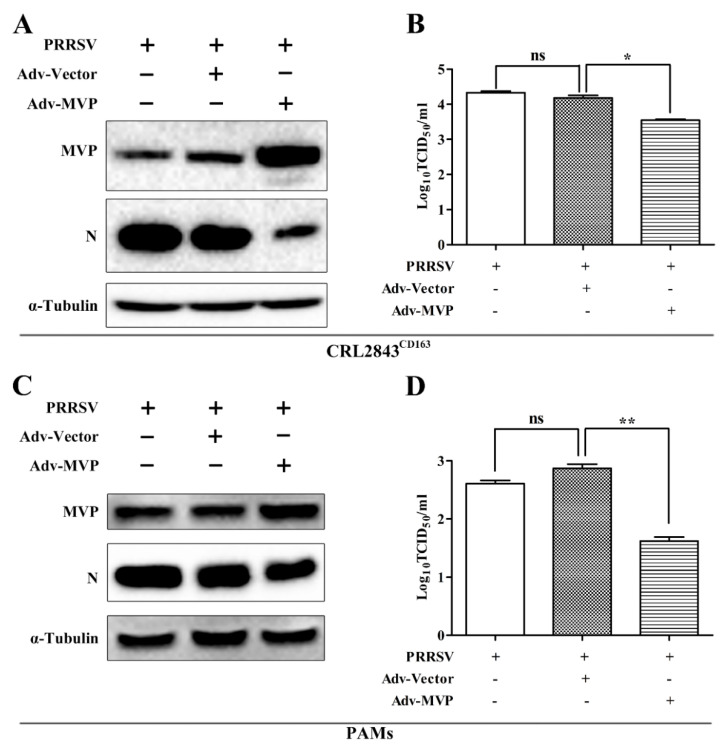
MVP overexpression inhibited PRRSV replication in CRL2843^CD163^ cells and PAMs. After infection with Adv–Vector or Adv–MVP (MOI = 5) for 24 h or 12 h, CRL2843^CD163^ cells or PAMs were infected with GD-HD PRRSV at an MOI of 1 or 0.1 for 48 h or 24 h, respectively. The protein expression levels of MVP and PRRSV N were detected by Western blot (**A**,**C**). Virus titers from cell supernatants were measured by TCID_50_ (**B**,**D**). The α-tubulin was used as internal loading controls in Western blot analysis. Data from three independent experiments are expressed as the means ± SD. The analysis of differences between the means was performed using ANOVA and is marked by ns (no significant), * (*p* < 0.05), and ** (*p* < 0.01).

The above results suggest that PRRSV infection could downregulate the expression of MVP, as shown in Figure 1. To assess the role of MVP in PRRSV infection, Adv–MVP was constructed and used to infect CRL2843^CD163^ cells to overexpress MVP. After infection with Adv–MVP or Adv–Vector, CRL2843^CD163^ cells were infected with the GD-HD PRRSV strain for 48 h. The results showed that the expression level of PRRSV N protein (Figure 2A) was significantly reduced by MVP overexpression when compared with the Adv–Vector control. Meanwhile, PRRSV progeny titers in cell culture supernatants decreased by 0.63 log_10_ with the overexpression of MVP (Figure 2B). Similarly, MVP overexpression also led to a decrease in levels of PRRSV N protein (Figure 2C) and progeny virus production (Figure 2D) in PAMs.

**Figure 3:**
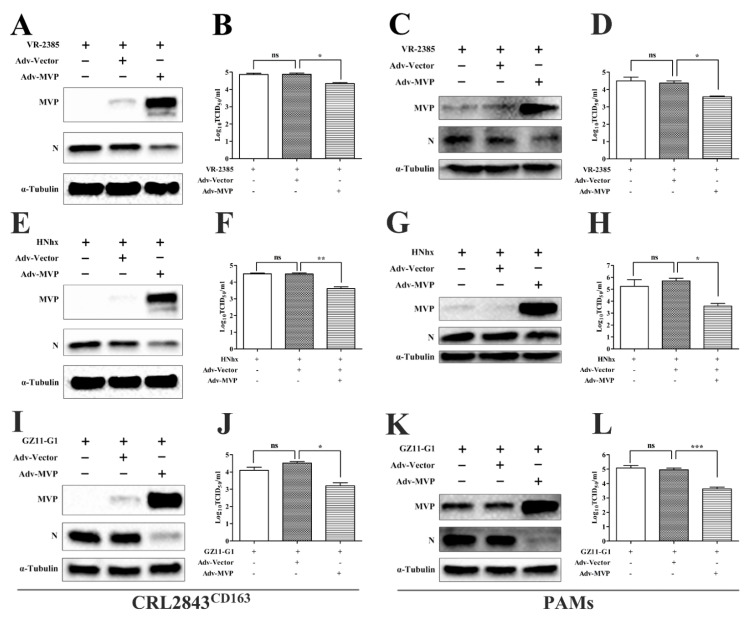
Inhibition effects of MVP on PRRSV replication are virus-strain-independent in CRL2843^CD163^ cells and PAMs. CRL2843^CD163^ cells or PAMs were infected with Adv−Vector or Adv−MVP (MOI = 5) for 24 h or 12 h, respectively. Then, CRL2843^CD163^ cells or PAMs were infected with PRRSV at an MOI of 1 or 0.1 for 48 h or 24 h, respectively. The protein expression levels of MVP and PRRSV N were detected by Western blot (**A**,**E**,**I**,**C**,**G**,**K**). Virus titers from cell supernatants were measured by TCID_50_ (**B**,**F**,**J**,**D**,**H**,**L**). The α-tubulin was used as internal loading controls in Western blot analyses. Data from three independent experiments are expressed as the means ± SD. The analysis of differences between the means was performed using ANOVA and is marked by ns (no significant), * (*p* < 0.05), ** (*p* < 0.01) and *** (*p* < 0.001).

To test whether or not the MVP-induced anti-PRRSV effect was strain-dependent, one PRRSV-1 isolate and two PRRSV-2 isolates were used to evaluate the antiviral activity of MVP in CRL2843^CD163^ cells and PAMs. The N protein level (Figure 3A,C,E,G,I,K) and progeny titers (Figure 3B,D,F,H,J,L) of the two types of PRRSV were decreased. These results suggested that the inhibition effect of MVP on PRRSV replication was not strain-dependent.

### 3.3. Knockdown of MVP Promotes PRRSV Replication In Vitro

**Figure 4:**
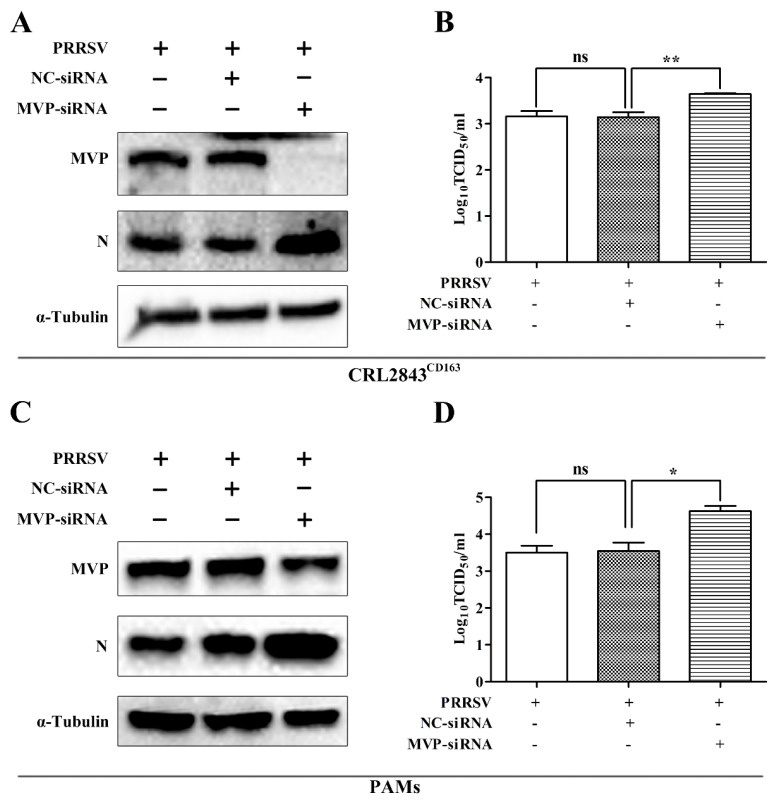
MVP knockdown promoted PRRSV replication in CRL2843^CD163^ cells and PAMs. CRL2843^CD163^ cells or PAMs were transfected with NC−siRNA or MVP−siRNA for 12 h or 36 h followed by infection with GD-HD PRRSV at an MOI of 1 or 0.1 for 48 h or 24 h, respectively. The protein expression levels of MVP and PRRSV N were detected by Western blot (**A**,**C**). Virus titers in cell supernatants were measured by TCID_50_. (**B**,**D**) The α-tubulin was used as an internal loading control in Western blot analysis. Data from three independent experiments are expressed as the means ± SD. The analysis of differences between the means was performed using ANOVA and is marked by ns (no significant), * (*p* < 0.05), and ** (*p* < 0.01).

To evaluate the role of MVP in PRRSV infection from another angle, CRL2843^CD163^ cells were transfected with MVP–siRNA for MVP knockdown. Then, CRL2843^CD163^ cells were infected with the GD-HD PRRSV strain. At 48 hpi, MVP protein level was decreased by MVP–siRNA transfection, and an increase in PRRSV N protein level (Figure 4A) was observed. Moreover, the progeny virus yields increased by 0.5 log_10_ due to MVP knockdown (Figure 4B). In addition, PAMs, the target cells of PRRSV in vivo, were also used to assess the effect of MVP knockdown in PRRSV infection. The results for PAMs were similar to those in CRL2843^CD163^ cells. MVP knockdown also caused an increase in PRRSV N protein (Figure 4C) and progeny virus production levels (Figure 4D).

### 3.4. MVP Knockdown Partially Reverses the Inhibitory Effect of MVP Overexpression on PRRSV Replication

**Figure 5:**
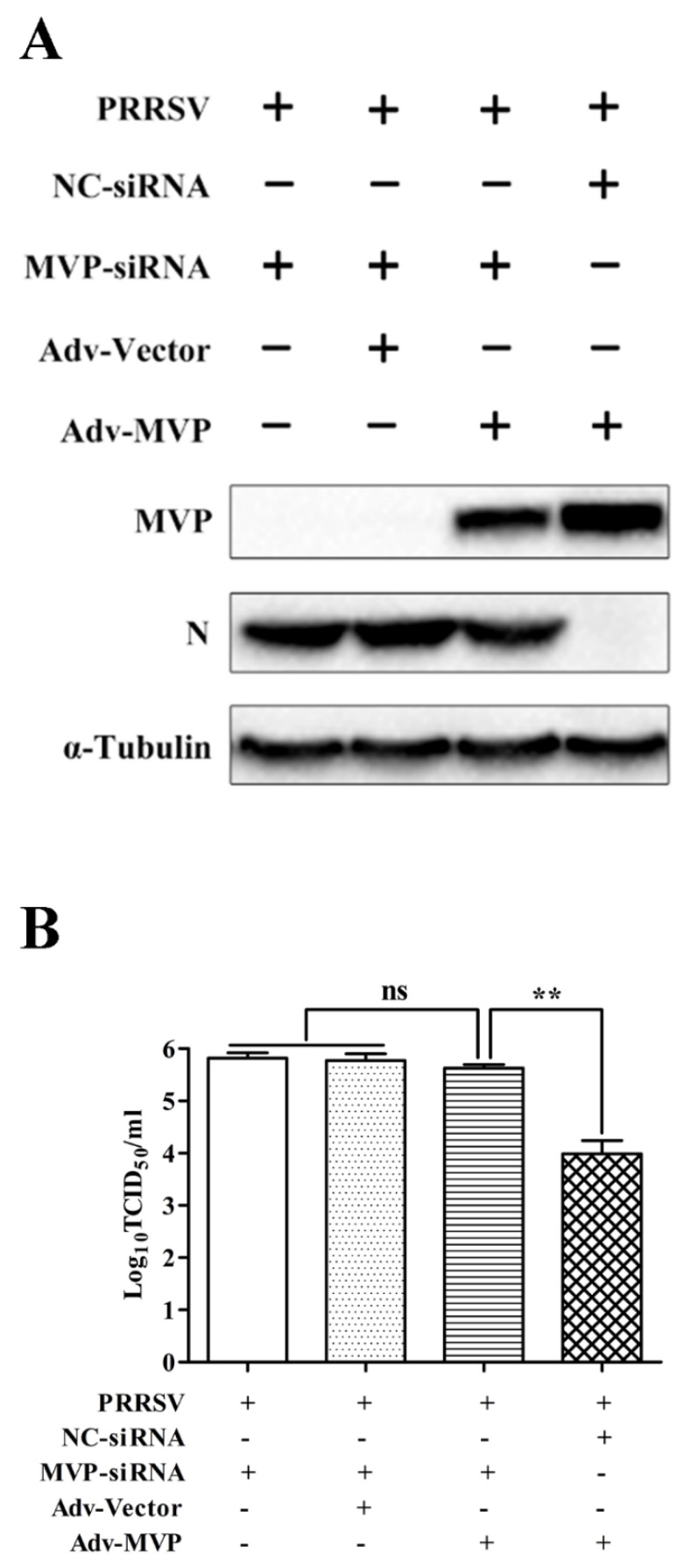
MVP knockdown has a restraining function on the inhibiting effect of MVP overexpression on PRRSV replication. CRL2843^CD163^ cells were transfected with NC−siRNA or MVP−siRNA for 12 h followed by infection with Adv−Vector or Adv−MVP (MOI = 5) for 12 h. Then, cells were infected with GD-HD PRRSV (MOI = 1) for 48 h. The expression levels of MVP and the PRRSV N protein were detected by Western blot (**A**). Virus titers in cell supernatants were measured by TCID_50_ (**B**). Alpha-tubulin was used as the internal loading control for Western blot analysis. Data from three independent experiments are expressed as the means ± SD. Analysis of differences between the means was performed using Student’s *t*-tests and ANOVA. Significant differences are marked by ns (no significant), and ** (*p* < 0.01) and.

The role of MVP in PRRSV replication was confirmed by MVP overexpression and MVP knockdown. To further verify the effect of MVP on PRRSV replication, CRL2843^CD163^ cells were transfected with MVP–siRNA before Adv–MVP and PRRSV infection. Compared to the controls pretreated with NC–siRNA, the cells pretreated with MVP–siRNA showed increased levels of PRRSV N protein (Figure 5A) and progeny virus production (1.64 log_10_) (Figure 5B). These results confirmed that MVP knockdown partially reversed the inhibitory effects of MVP overexpression, suggesting that MVP expression has a direct effect on PRRSV replication.

### 3.5. MVP Induces the Expression of Type Ⅰ IFNs in PRRSV-Infected PAMs

**Figure 6:**
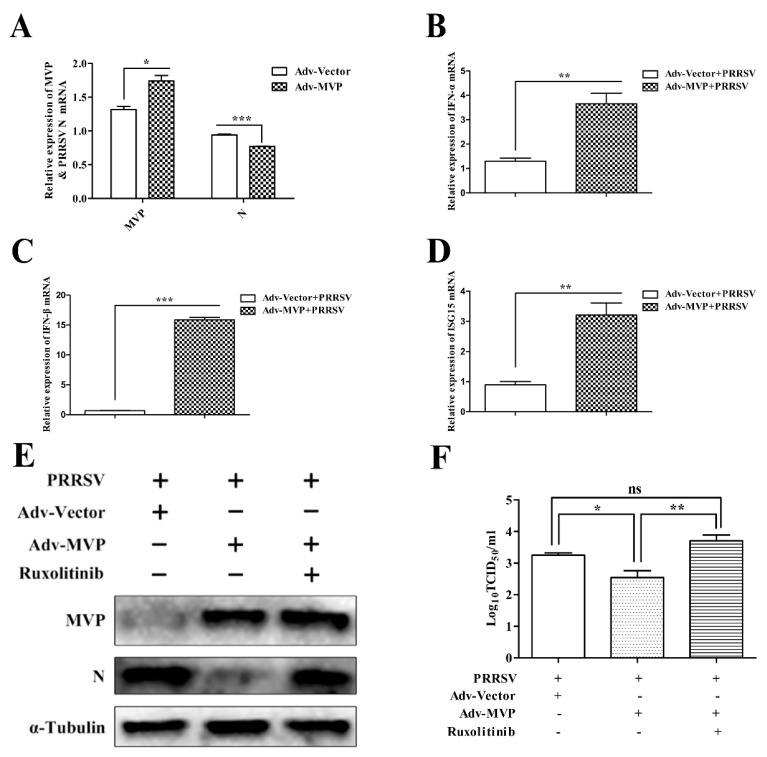
The anti-PRRSV activity of MVP was mediated by type Ⅰ IFNs. PAMs were infected with Adv−Vector or Adv−MVP (MOI = 5) for 12 h. Subsequently, cells were infected with GD-HD PRRSV (MOI = 0.1) for 24 h. The mRNA expression levels of MVP, PRRSV N (**A**), IFN-α (**B**), IFN-β (**C**) and ISG15 (**D**) were detected by qPCR. GAPDH was used as an internal loading control in qPCR analysis. (**E**,**F**) PAMs were infected with Adv−Vector or Adv−MVP (MOI = 5) for 2 h and subsequently treated with ruxolitinib (5 μM) for 12 h. After infection with GD-HD PRRSV (MOI = 0.1) for 24 h, the protein expression levels of MVP and PRRSV N were detected by Western blot (**E**). Virus titers from cell supernatants were measured by TCID_50_ (**F**). The α-tubulin was used as an internal loading control in Western blot analysis. Data from three independent experiments are expressed as the means ± SD. Analysis of differences between the means was performed using Student’s *t*-tests or ANOVA and is marked by ns (no significant), * (*p* < 0.05), ** (*p* < 0.01) and *** (*p* < 0.001).

To explore the mechanism of action of MVP on the inhibition of PRRSV replication, PAMs were infected with Adv–MVP or Adv–Vector before PRRSV infection. The mRNA levels of MVP, PRRSV N, IFN-α, IFN-β and interferon-stimulated gene 15 (ISG15) were measured by qPCR. As shown in Figure 6A–D, in PRRSV-infected cells, the expression levels of IFN-α, IFN-β and ISG15 mRNA were increased when MVP was overexpressed. Therefore, the MVP–mediated anti-PRRSV state was activated by inducing the expression of type Ⅰ IFNs.

To further confirm that type I IFNs are responsible for the anti-PRRSV effect of MVP, a (JAK) 1/2 inhibitor, ruxolitinib, was used to test if it reversed the effect of MVP overexpression on PRRSV replication. PAMs were treated with ruxolitinib after Adv–MVP or Adv–Vector infection. Subsequently, PAMs were infected with the GD-HD PRRSV strain. Compared with the Adv–MVP control, the N protein level (Figure 6E) and progeny titers (Figure 6F) were increased by ruxolitinib. The results strengthened the conclusion that the inhibitory effect of MVP overexpression induced the synthesis of type Ⅰ IFNs.

## 4. Discussion

It has previously been reported that several viruses enhance MVP expression in their corresponding susceptible cell lines. In the abovementioned experiments, the infectious dose and/or incubation time of each virus was not identical. In this study, MVP mRNA and protein levels were inhibited in PRRSV-infected PAMs and CRL2843^CD163^ cells at 24 and 48 hpi. The reason for these opposing results may be associated with the ability of the virus to induce type Ⅰ IFNs in different cells. For example, in the case of influenza A virus, the expression of type Ⅰ IFNs was induced in influenza A virus-infected MDCK cells, but this was not enough to inhibit virus replication [46]. However, type Ⅰ IFNs were not produced in PAMs after infection by PRRSV [47,48]. Additionally, CRL2843^CD163^ cells only have one PRRSV receptor. In addition, CD163, CD151, CD169, CD209, heparan sulphate, vimentin and non-muscle myosin heavy chain 9 (MYH9) also have been identified as PRRSV receptors [49,50]. Compared with PAMs, the infection efficiency of PRRSV on CRL2843^CD163^ cells is significantly lower. Therefore, in investigating the variance of MVP expression in PRRSV infection, CRL2843^CD163^ cells were infected with GD-HD PRRSV at an MOI of 10 when PAMs were infected with GD-HD PRRSV at an MOI of 1.

Overexpression and knockdown methods are often used to evaluate the role of a protein during virus infection in vitro. Until now, only one study has reported that MVP overexpression inhibits the replication of several viruses in vitro, including HCV, VSV, IAV and EV71 [40]. To investigate whether MVP has an anti-PRRSV capability, adenovirus–mediated MVP overexpression or siRNA–mediated MVP knockdown was conducted in CRL2843^CD163^ cells and PAMs. Of additional note, PRRSV in circulation in China is composed of both PRRSV-1 and PRRSV-2 [51]. For this reason, PRRSV-1 and PRRSV-2 strains were used to test the effect of MVP overexpression. Our data showed that MVP overexpression inhibited PRRSV replication in CRL2843^CD163^ cells and PAMs. The anti-PRRSV role of MVP was further confirmed using siRNA gene knockdown. Knockdown of endogenous MVP facilitated PRRSV replication in CRL2843^CD163^ cells and PAMs. Furthermore, the inhibitory effect of MVP overexpression on PRRSV replication was reversed by the knockdown of MVP. These results indicated that MVP is a host component with anti-PRRSV capabilities.

Type Ⅰ IFNs are crucial effector cytokines that play an important protective role during viral infection. The production of type Ⅰ IFNs is induced by pathogen-associated molecular patterns that recognize pattern-recognition receptors on sentinel cells in the innate immune system. The initiation of signaling of type Ⅰ IFNs is mediated by interacting with their receptors on the surface of cells. After type Ⅰ IFNs bind to their receptors, JAK is activated and STATs are phosphorylated. IFN-stimulated genes (ISGs) are induced following the phosphorylation of STATs, which mediate inhibition of the viral replicative cycle [52]. IFN-α and IFN-β are the most common and well-defined type Ⅰ IFNs subtypes [53]. Previous research has demonstrated that MVP overexpression induces an intracellular antiviral response by upregulating the production of IFN-α and IFN-β. In addition, MVP is downstream of TLR signaling pathways and leads to type Ⅰ IFN production in virus infection [40]. In pigs, the type Ⅰ IFN family consists of IFN-α, IFN-αω, IFN-β, IFN-δ, IFN-ε, IFN-κ and IFN-ω [54]. PRRSV inhibits type Ⅰ IFN induction by interfering with retinoic-acid-inducible protein I (RIG-I) and Toll-like receptor (TLR3) signaling pathways. Furthermore, non-structural protein 1 (nsp1) of PRRSV, as well as nsp2, nsp4 and nsp11, have been demonstrated to be type Ⅰ IFN antagonists by inhibiting the activation of interferon regulatory factor 3 (IRF3) and/or nuclear factor kappa-B (NF-κB) [5,55]. Except for the inhibition of type I IFN induction in PRRSV infection, PRRSV could also inhibit IFN-activated JAK-STAT signaling [56]. In addition, recombinant porcine IFN-α/IFN-β was proven to inhibit PRRSV replication [57,58]. In this study, our results showed that Adv–mediated MVP overexpression induced the production of IFN-α, IFN-β and ISG15 in PRRSV-infected PAMs. Moreover, (JAK) 1/2 inhibitor ruxolitinib blocked the anti-PRRSV effects of MVP overexpression, which was further evidence that MVP could induce the expression of type I IFNs to inhibit PRRSV replication.

## 5. Conclusions

In summary, this study demonstrates the effect of MVP on PRRSV infection in vitro. As a host agent, the expression of MVP was regulated during PRRSV infection. MVP inhibits PRRSV replication in CRL2843^CD163^ cells and PAMs, and induces the anti-PRRSV innate immune response. All the results suggest that MVP could be used as a potential regulatory target to prevent and treat PRRSV.

## Figures and Tables

**Table 1:** The sequences of primers used in this study for real-time RT-PCR.

Gene	Primer	Sequence (5′→3′)
MVP	Forward primer	ACACTTCTGGATACAGGTGAGC
	Reverse primer	AGTCTTCGGTCCAACCTCCA
PRRSV ORF7	Forward primer	AAACCAGTCCAGAGGCAAGG
	Reverse primer	GCAAACTAAACTCCACAGTGTAA
IFN-α	Forward primer	GCCTCCTGCACCAGTTCTACA
	Reverse primer	TGCATGACACAGGCTTCCA
IFN-β	Forward primer	TGCAACCACCACAATTCC
	Reverse primer	CTGAGAATGCCGAAGATCTG
ISG15	Forward primer	TCCTGGGCTCTAGGAGCTTT
	Reverse primer	ATGCCATCATGCAGTCCCTC
GAPDH	Forward primer	CCTTCCGTGTCCCTACTGCCAAC
	Reverse primer	GACGCCTGCTTCACCACCTTCT

## Data Availability

All available data are presented in the article.
